# A fossil protein chimera; difficulties in discriminating dinosaur peptide sequences from modern cross-contamination

**DOI:** 10.1098/rspb.2017.0544

**Published:** 2017-05-31

**Authors:** Michael Buckley, Stacey Warwood, Bart van Dongen, Andrew C. Kitchener, Phillip L. Manning

**Affiliations:** 1 Faculty of Science and Engineering, The University of Manchester, Manchester Institute of Biotechnology, Manchester M1 7DN, UK; 2 School of Earth and Environmental Sciences, Faculty of Science and Engineering, Interdisciplinary Centre for Ancient Life, The University of Manchester, Manchester, M13 9PL, UK; 3 Faculty of Biology, Medicine and Health, The University of Manchester, Michael Smith Building, Manchester M13 9PL, UK; 4 Department of Natural Sciences, National Museums Scotland, Chambers Street, Edinburgh EH1 1JF, UK; 5 Institute of Geography, School of Geosciences, University of Edinburgh, Drummond Street, Edinburgh EH8 9XP, UK; 6 Department of Geology and Environmental Geosciences, College of Charleston, 66 George Street, Charleston, SC 29424, USA

**Keywords:** ancient collagen, dinosaur protein, ostrich, palaeoproteomics, *Tyrannosaurus*, *Brachylophosaurus*

## Abstract

A decade ago, reports that organic-rich soft tissue survived from dinosaur fossils were apparently supported by proteomics-derived sequence information of exceptionally well-preserved bone. This initial claim to the sequencing of endogenous collagen peptides from an approximately 68 Myr *Tyrannosaurus rex* fossil was highly controversial, largely on the grounds of potential contamination from either bacterial biofilms or from laboratory practice. In a subsequent study, collagen peptide sequences from an approximately 78 Myr *Brachylophosaurus canadensis* fossil were reported that have remained largely unchallenged. However, the endogeneity of these sequences relies heavily on a single peptide sequence, apparently unique to both dinosaurs. Given the potential for cross-contamination from modern bone analysed by the same team, here we extract collagen from bone samples of three individuals of ostrich, *Struthio camelus*. The resulting LC–MS/MS data were found to match all of the proposed sequences for both the original *Tyrannosaurus* and *Brachylophosaurus* studies. Regardless of the true nature of the dinosaur peptides, our finding highlights the difficulty of differentiating such sequences with confidence. Our results not only imply that cross-contamination cannot be ruled out, but that appropriate measures to test for endogeneity should be further evaluated.

## 1. Introduction

The search for ancient biomolecules from deep-time fossils has led to some exceptional claims regarding the preservation of organics within fossilized material. In particular, Schweitzer *et al.* [1] reported on the startling discovery of soft-tissue preservation in the femur of a *Tyrannosaurus rex*, apparently preserved for over 68 Myr, a length of time vastly greater than thought possible given the experimentally measured rates of decay for the component structural proteins, particularly collagen [2]. With exceptional preservation of organic molecules comes the possibility of retrieving a wealth of genetic information from periods of time long before our presence on the Earth. However, it has long been accepted that morphological preservation does not imply molecular preservation [3]. Yet in 2007, the same team reported on the sequencing of collagen that they reported to be endogenous to *T. rex* specimen MOR 1125 [4,5]. Obtaining molecular-sequence information would be the gold standard for supporting such claims of soft-tissue survival, but confirming the authenticity of ancient biomolecule sequences is difficult [6]. By contrast, Manning *et al.* [7] reported on the exceptionally preserved remains of a hadrosaur from the Hell Creek Formation (USA), which included mineralized skin, tendon and associated skeletal material, but even from such an exceptionally preserved specimen, they were only able to demonstrate the presence of protein breakdown products using total amino acid analyses, pyrolysis gas chromatography mass spectrometry (Py-GCMS), Fourier transform infrared (FTIR) spectroscopy, matrix assisted laser desorption ionization time of flight (MALDI-TOF) mass spectrometric peptide mass fingerprinting and proteomic analyses [7].

### (a) *Tyrannosaurus rex* collagen sequences

In the case of the *T. rex* specimen (MOR 1125), these initial studies were supported by two main lines of supporting molecular evidence: immunological data and sequence information [1]. This first choice of support is no longer widely accepted as ideal for such claims, given that immunological techniques have been shown to yield false-positive results [8]. The authenticity of any findings based on this approach rests solely on sequence interpretation. In its first release, this was fraught with multiple incorrect post-translational modification (PTM) assignments in the form of hydroxylated glycine residues [5]; a clear indication of the potential problems is the reliance on probability-based matching algorithms of current proteomics-based techniques. In direct response to this first report, several criticisms arose related to potential forms of contamination [6,9,10] or statistical artefact relating to such a proteomics approach [11].

On the grounds that mineralized and non-mineralized coatings have been found extensively in the porous trabecular bone of a variety of vertebrate fossils across time, including dinosaurs, Kaye *et al.* [10] proposed that the *T. rex* specimen was likely similarly contaminated with bacterial biofilm, thus explaining the morphological similarity to the blood vessels and osteocytes that they attack. In addition, the blood-cell-like iron–oxygen spheres found in the vessels were identified as an oxidized form of formerly pyritic framboids. Interestingly, similar thin linings on Haversian canals within apatite were identified by infrared and electron microprobe analysis of ossified tendon by Manning *et al.* [7] and clearly showed preserved mineral zonation, with silica and trapped carbon dioxide. FTIR analysis of the tendon showed clear structural control of organic compounds within the Haversian canals, suggesting that organic material may have persisted. However, this study concluded that the organic signal may have been associated with breakdown products of the original biomaterial deposited within the tendon, consistent with the presence of the endogenous breakdown products of organic material identified from other regions of the specimen but not able to yield any such sequence information [7].

Bern *et al.* [9] reanalysed the original *T. rex* sequence data to infer that the sample was predominantly laboratory contaminants, soil bacteria and bird-like haemoglobin and collagen. They suggested that of the six peptides that Asara *et al.* [5] deposited in GenBank (GATGAPGIAGAPGFPGARGAPGPQGPSGAPGPK, GSAGPPGATGFPGAAGR, GVQGPPGPQGPR, and GVVGLPGQR from collagen alpha-I type I, GLVGAPGLRGLPGK from collagen alpha-1 type II and GLPGESGAVGPAGPIGSR from collagen alpha-2 type I), only the first three of these could be considered statistically significant, calling for the latter to be dropped from GenBank. However, despite the unexpected presence of haemoglobin, a protein only typically seen in relatively recent samples [12,13], the presence of the remaining collagen peptides was not accepted as being contamination for reasons that remain unclear.

### (b) *Brachylophosaurus canadensis* collagen sequences

Following the initial 2007 report [5], the same team reported similar collagen peptide sequence matches from a hadrosaurine dinosaur, an approximately 78 Ma *Brachylophosaurus canadensis* (MOR 2598; table 1) [14]. However, although it had already been suggested that standards be set in place, like those for the field of ancient DNA, this second study once again aimed to rely on an immunological approach as the main line of support, despite the ability to record chemical decay within proteins through PTMs such as oxidations and deamidations [15–17], or even a range of others identified by the same team in ancient moa [18].

**Table 1.** Peptide matches to *Brachylophosaurus* collagen from Schweitzer *et al.* [14] and the taxa they can be found in as originally stated (with added observations by BLAST search in parentheses, with an emphasis on the potential contaminants). Underlined residues indicate post-translational modification (oxidation of P; deamidation of N).

peptide sequence	protein	taxa
GLTGPIGPPGPAGAPGDKGEAGPSGPPGPTGAR	COL1A1	ostrich and mammals
GSAGPPGATGFPGAAGR	COL1A1	*Tyrannosaurus rex*, chicken and mammals (and others, including alligator^a^)
GATGAPGIAGAPGFPGAR	COL1A1	*Tyrannosaurus rex*, chicken, alligator and amphibians (and other reptiles and birds)
GETGPAGPAGPPGPAGAR	COL1A1	chicken (and other birds, including ostrich)
GVQGPPGPQGPR	COL1A1	*Tyrannosaurus rex*, chicken, alligator and opossum (and others, including ostrich)
GPSGPQGPSGAPGPK	COL1A1	chicken, alligator, rat and opossum (and others, including ostrich)
GSNGEPGSAGPPGPAGLR	COL1A2	chicken and alligator (and other birds, including ostrich)
GLPGESGAVGPAGPP*GSR	COL1A2	*Tyrannosaurus rex*

*Note that this amino acid sequence (bold) was ‘corrected’ from the *T. rex* sequence originally proposed by Asara *et al.* [5].

^a^This part of the sequence is not currently complete for ostrich (*Struthio*) by BLAST search.

Following Schweitzer *et al.* [14], there have been no further published attempts to verify the endogeneity of either published samples of purported dinosaur collagen sequences from other research groups, despite the lack of potential means to clarify the extent of decay within the proteins, of which we would expect substantial alteration [7]; members of the same team subsequently went on to report even more exceptional peptide matches to soft-tissue structures, in which they interestingly did report on the levels of deamidation and made clear attempts to separate modern from fossil material during the laboratory process [19]. The published record to date could be considered to lean in favour of endogeneity, with Peterson *et al.* [20] arguing against the microbial biofilm interpretation, suggesting that the crystallization of microbial biofilms on decomposing organic matter within vertebrate bone in early taphonomic stages may contribute to the preservation of primary soft tissues deeper in the bone structure [14].

A subsequent study mapping the molecular locations of the matched collagen peptides from both dinosaurs also implied that it was functionally significant regions of the collagen fibrils that were matched [21]. Although it was suggested that this non-random distribution could support the hypothesis that the peptides are produced from the extinct organisms, while also suggesting a chemical mechanism for survival, it does not rule out cross-contamination in which the same ‘mechanism for survival’ could equally apply to enhanced likelihood of contaminant peptides. More recently, a second collagen-based study has been published that placed further emphasis on the cleaning of the instrumentation used in addition to separate laboratories for extant and fossil material [22], presenting an overlapping set of peptides. Intriguingly, these do not include the peptide sequence found as unique to both dinosaurs (table 2). As a result, the phylogenetic analysis of this latest extraction places the *Brachylophosaurus* as sister-group to alligators as well [22], clearly highlighting concern regarding the limitations of the study to date. They do, however, all match with peptides from alligator type 1 collagen, a species concurrently analysed in their previous works as modern reference material [5,14] even if not necessarily contamination caused at the time of the most recent sampling.

**Table 2.** Peptide matches to *Brachylophosaurus* collagen from Schroeter *et al*. [22] and the taxa they can be found in by BLAST search in parentheses, with an emphasis on the potential contaminants. Underlined residues indicate post-translational modification (oxidation of P). The emboldened peptide is reported as having two hydroxylated prolines even though we routinely observe this peptide with only one (at P3), with a nearby A–S transition identified previously as being problematic to distinguish; they acknowledge in the electronic supplementary material that it could be either.

peptide sequence	protein	taxa
*GSAGPPGATGFPGAAGR	COL1A1	*Tyrannosaurus rex*, chicken, mammals (and others, including alligator)
*GATGAPGIAGAPGFPGAR	COL1A1	*Tyrannosaurus rex*, chicken, alligator and amphibians (and other reptiles and birds)
GFPGADGIAGPK **(GFPGADGIsGPK)**	COL1A1	ostrich and others (*alligator)
GFPGLPGPSGEPGK	COL1A1	alligator and ostrich (and others, ranging from fish to mammals)
GQAGVMGFPGPK	COL1A1	alligator and ostrich (and others)
EGPVGFPGADGR	COL1A2	alligator and ostrich (and others, including reptiles, birds and mammals)
GATGLPGVAGAPGLPGPR	COL1A2	alligator (and rodents)
GEPGNIGFPGPK	COL1A2	alligator and ostrich (and others, including birds and mammals)

*Note that these were the two peptides observed in both analyses [5].

Given that the only reports that appear to favour the most recent studies cannot rule out cross-contamination, we set out to test whether or not the reported set of unique collagen peptides (i.e. [5,14], excluding [22] as not containing unique peptides) could simply reflect cross-sample contamination from the modern reference material used; in this case, ostrich (*S. camelus*) bone (alligator was also used in the latter study, but not evaluated here in determining the unique dinosaur peptide because it was not used in the earlier study). In this study, we aimed to investigate the differences between sequences from ostrich bone collagen and those reported for both *T. rex* (MOR 1125) and *B. canadensis* (MOR 2598).

## 2. Material and methods

### (a) Proteomics analysis of modern ostrich bone

Three modern ostrich bone specimens were sampled, two (CC1254 and CC507) from Creswell Crags Heritage Centre (Derbyshire, UK) and one from our own collections (UM902) purchased from Ostrich Solutions (UK). Proteome extraction was solely restricted to our standard GuHCl-based approach following decalcification [13]. In brief, decalcification with 0.6 M hydrochloric acid (HCl) for approximately 18 h (overnight), and centrifuged at 14 000 r.p.m. for 5 min. The supernatant was removed and frozen, while the acid-insoluble residue was gelatinized with 6 M guanidine hydrochloride/5 mM Tris–HCl for a further 18 h. The acid-soluble collagen was applied to a 10 kDa ultrafilter (Vivaspin, UK) and centrifuged, which was repeated with the centrifuged supernatant from the acid-insoluble residue extraction. Once the acid-soluble proteins had passed through the ultrafilter, two volumes of ammonium bicarbonate (50 mM; ABC) were also passed through. Once both volumes had filtered through, a further 200 µl of ABC were added to the filter, mixed and recovered. This was incubated with 10 µl 100 mM dithiothreitol (in 50 mM ABC) for 10 min at 60°C. After cooling, 40 µl of iodoacetamide were added to each sample and stored in the dark at room temperature for 45 min. A further 10 µl 100 mM dithiothreitol were added to quench the reaction and the sample digested overnight with 2 µg sequencing grade trypsin (Promega, UK) at 37°C. The tryptic digests were cleaned using C18 ziptips following manufacturer's protocol (Varian OMIX, UK), dried down and resuspended with 10 µl 5% acetonitrile/0.1% formic acid.

### (b) Liquid chromatography–mass spectrometry/mass spectrometry analyses

Digested samples were analysed by LC–MS/MS using an UltiMate^®^ 3000 Rapid Separation LC (RSLC, Dionex Corporation, Sunnyvale, CA, USA) coupled to an Orbitrap Elite (Thermo Fisher Scientific, Waltham, MA, USA) mass spectrometer (120 k resolution, Full Scan, Positive mode, normal mass range mass-to-charge ratio (*m/z*) 350–1500). Peptides in the sample were separated on a 75 mm × 250 µm i.d. 1.7 µM ethylene bridged hybrid (BEH) C18 analytical column (Waters, UK) using a gradient from 92% A (0.1% formic acid in water) and 8% B (0.1% formic acid in acetonitrile) to 33% B in 44 min at a flow rate of 300 nl min^−1^. Peptides were automatically selected for fragmentation by data-dependent analysis; six MS/MS scans (Velos ion trap, product ion scans, rapid scan rate, Centroid data; scan event: 500 count minimum signal threshold, top six) were acquired per cycle, dynamic exclusion was employed and one repeat scan (two MS/MS scans total) was acquired in a 30 s repeat duration, with that precursor being excluded for the subsequent 30 s (activation: collision-induced dissociation (CID), 2+ default charge state, 2 *m/z* isolation width, 35 eV normalized collision energy, 0.25 Activation Q, 10.0 ms activation time).

In addition to the above, one ostrich bone proteome digest was also analysed using high resolution in the MS/MS to demonstrate the ability to resolve sequence ambiguity of the homologous ostrich peptide unique *T-rex* peptide (GPP(Oxidation)GESGAVGPAGPIGSR versus GLPGESGAVGPAGPP(Oxidation)GSR, respectively). This was done by employing a method in which peptides were automatically selected for fragmentation by data-dependent analysis; and performing six MS/MS scans. However, in this instance, three MS/MS scans were low-mass accuracy CID scans and three were high-mass accuracy higher energy collisional dissociation (HCD) scans. Each precursor that was first selected for CID fragmentation was then selected for HCD fragmentation. As described above, the CID spectra were acquired in the Velos ion trap, with the same parameters as above. HCD spectra were acquired in the Orbitrap, with a mass resolution of 15 k. All other parameters were as with the Velos, except no selection is made for Activation Q.

### (c) Database searching

Peptide spectra obtained via LC–MS/MS were searched against the SwissProt database for matches to primary protein sequences using the Mascot search engine (v. 2.2.0.6; Matrix Science, London, UK). Each search included the fixed carbamidomethyl modification of cysteine (+57.02 Da) and the variable modifications for asparagine and glutamine deamidation (+0.98 Da), serine and threonine phosphorylation (+79.99 Da) and oxidation of lysine, proline and methionine residues (all +15.99 Da) to account for PTMs and diagenetic alterations (the oxidation of lysine and proline being equivalent to hydroxylation commonly observed in collagen, the dominant protein in bone). Enzyme specificity was limited to trypsin (trypsin/P) with up to two missed cleavages allowed, mass tolerances were set at 5 ppm for the precursor ions and 0.5 Da for the fragment ions; all spectra were considered as having either 2+ or 3+ precursors and the peptide ion score cut-off was set at 30 for more confident matches. Repeat searches were also carried out using Error Tolerant search parameters with only one missed cleavage and the carbamidomethyl fixed modifications, and oxidations of both lysine and arginine selected to ensure that collagen would be adequately matched. These were carried out against the SwissProt database to retain similar search conditions to those available to the original studies, despite more avian and reptilian sequences being available elsewhere.

## 3. Results

### (a) Modern ostrich bone collagen matches

In our analyses of modern ostrich bone samples, which were analysed several months/years apart and from three distinct individuals, there were unequivocal 100% sequence matches both for the *Brachylophosaurus* and *Tyrannosaurus* uploaded to SwissProt (table 3). In almost every case the Mascot score was relatively high, whereby even the ‘unique dinosaur peptide’ GLPGESGAVGPAGPPGSR was identified with all scores 70 or above (although not directly comparable, higher than the originally reported Mascot score of 54.3), despite this not being reported in the ostrich bone collagen analyses by Asara *et al.* [5].

**Table 3.** Mascot search result scores of digested proteome extracts from three different ostrich bone specimens analysed months apart and the similarity to the ostrich (*Struthio*) sequence as a percentage. Note that the first tryptic peptide of the emboldened sequence was also matched in every sample without the missed cleavage at the K residue. Underlined residues indicate post-translational modification (oxidation of P/K; deamidation of N); scores in parentheses represent higher scores with deamidated peptides or with one additional oxidation. *m/z*, mass-to-charge ratio.

	peptide sequence	*m/z*	CC1254	CC507	UM902	similarity (%)
BrachyA1/TrexA1	GVQGPPGPQGPR	1161.6	57	53 (60)	59 (65)	100
BrachyA1	GPSGPQGPSGAPGPK	1305.6	71	74	53 (80)	100
BrachyA1/TrexA1	GSAGPPGATGFPGAAGR	1458.7	94	120	123	100
BrachyA1	GETGPAGPAGPPGPAGAR	1531.7	84	85	66	100
BrachyA1	GLTGPIGPPGPAGAPGDK	1589.8	58	75	45	100
BrachyA1	**GLTGPIGPPGPAGAPGDKGEAGPSGPPGPTGAR**	2878.4	45	45	36 (44^a^)	100
BrachyA2/TrexA2	GLPGESGAVGPAGPPGSR^b^	1577.8	70	76	78	89
BrachyA2	GSNGEPGSAGPPGPAGLR	1608.7	80 (93)	58 (79)	71	100
BrachyA1/TrexA1	GATGAPGIAGAPGFPGAR	1571.8	74	73	58	100
TrexA1	GAPGPQGPSGAPGPK	1305.6	69	62	46 (71)	100
TrexA1	GVVGLPGQR	897.5	50	46	50	100

^a^Score with one additional oxidation.

^b^Peptide was considered unique to *T. rex* but see electronic supplementary material, S1–S6.

The only peptide that appears unique to the dinosaurs is the peptide at *m/z* 1577.7. However, the homologous sequence in chicken, *Gallus gallus domesticus*, is almost identical in terms of mass (GLPGESGAVGPAGPP(Oxidation)GSR in *T. rex*), where the proposed hydroxylation of the P_15_ is an isoleucine in chicken, which it was originally identified as in the *T. rex* specimen by Asara *et al.* [5]. In their later publication [14], they claimed this peptide as deriving from a similar but uniquely dinosaur peptide, emphasizing that their high-resolution instruments were capable of distinguishing between the two residues (high-resolution instruments should be capable of the separation, e.g. Ile monoisotopic mass 131.094635, Hyp 131.058243; difference 0.036392). However, this distinction would still require a near complete ion series particularly at both ends of the peptide, but their data for this peptide are not shown. In this case, their later interpretation that ‘hydroxyproline is more accurate than isoleucine/leucine’ is likely true, but its placement at position 15 may not necessarily be well supported without showing this ‘unique’ dinosaur peptide spectrum.

**Figure 1.**
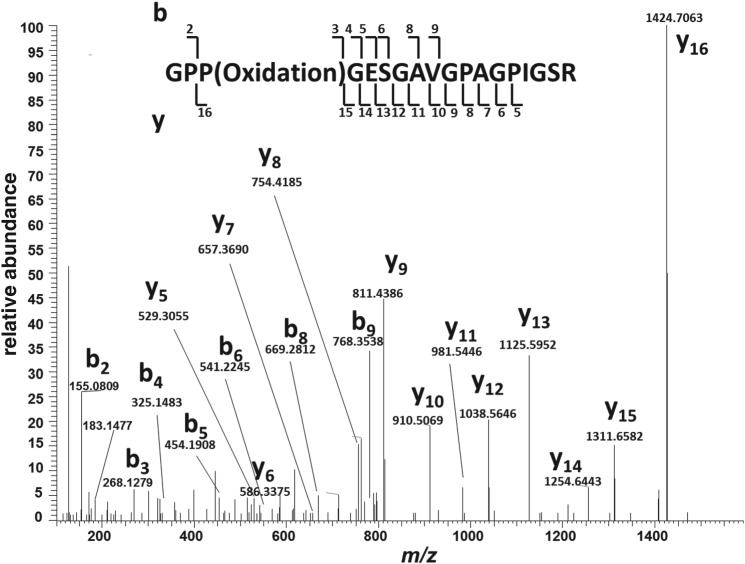
Tandem mass spectrum from high-resolution (HCD) fragmentation analysis of the peptide sequence (GPPGESGAVGPAGPIGSR) matched from our analysis of ostrich bone collagen that is homologous to the peptide proposed as unique to *T. rex* and *B. canadensis.*

**Figure 2.**
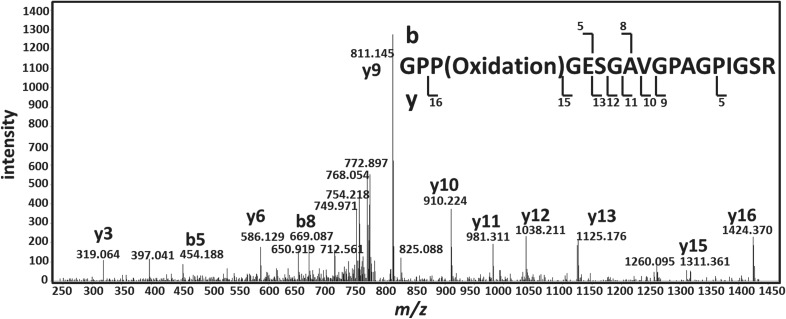
Tandem mass spectrum of the peptide sequence claimed as being endogenous to both dinosaurs with sequence (GLPGESGAVGPAGPPGSR) downloaded from the *B. canadensis* analysis by Schweitzer *et al.* [14].

Interestingly, we observe a subtle difference in the sequence for this peptide, at least in ostrich collagen regardless of the actual sequence identity of the dinosaur specimens (to which the ostrich confidently matches). From both the low-mass accuracy (CID) and high-mass accuracy (HCD) spectra, it is clear that the sequence identity at residues 1–3 as GPP(Oxidation)- can be readily determined, as indicated by the y_16_/b_2_ fragment ion pair (figure 1). However, typically determining the sequence identity at residue 15 in such an instance (i.e. distinguishing between an oxidated proline and an isoleucine/leucine residue) is not as straightforward because the mass difference is much smaller (at 75 ppm for y_4_). Here, we demonstrate that it can be done by acquiring an appropriate HCD spectrum for the precursor in question, i.e. at *m/z* 789.8972, and comparing the observed fragment ion masses with those calculated for the two carboxy-terminal peptide sequences in question (table 4). The mass differences between the observed values and those calculated for peptide sequence GLPGESGAVGPAGPP(Oxidation)GSR were found to be in the region 22.4–140.6 ppm, and outside the accuracy determined for the analyses. However, those for peptide sequence GPP(Oxidation)GESGAVGPAGPIGSR were between 0.5 and 11.6 ppm (table 4) demonstrating how it is possible to confirm the identity of the peptide (note that the precursor *m/z* observed for this peptide was identical to three decimal places to that observed for the homologous *T. rex* peptide). Interestingly, when we download the spectrum of the unique dinosaur peptide (100407RHad062807ndzipCID.2654.2654.2) from the published *Brachylophosaurus* dataset and search it against SwissProt using Mascot, the only match is to the peptide sequence (GPP(Oxidation)GESGAVGPAGPIGSR; score 50/expect value 0.0067). What is most telling is the absence of the expected y ion peak at *m/z* 1408.7 in their own spectrum (figure 2), but dominance of the peak at *m/z* 1424.4, as we would expect for the ostrich sequence (figure 1). However, regardless of the true sequences of the dinosaur peptides, our finding of 100% match to all sequences for both dinosaurs, including this variant sequence, highlights the difficulty of separating such sequences with confidence without clearly identifying the appropriate parts of the MS/MS spectra.

**Table 4.** The *m/z* values for detected fragment y ions for the peptide at 789.898 selected for HCD fragmentation. The observed value is given, along with the calculated values for each fragment according to the two sequences (*T. rex* and *Struthio*) under question, emphasizing the ability to distinguish between an oxidated proline and isoleucine/leucine residue in the carboxy-terminal region of the peptide.

Frag ion	observed	P(Oxidation)- L	ppm	L-P(Oxidation)	ppm	ppm calc
y_4_	—	432.2565	—	432.2201	—	75
y_5_	529.3055	529.3093	7.2	529.2729	61.6	69
y_6_	586.3375	586.3307	11.6	586.2944	73.5	62
y_7_	657.369	657.3678	1.8	657.3315	57	55
y_8_	754.4185	754.4206	2.8	754.3842	45.5	48
y_9_	811.4386	811.4421	4.3	811.4057	40.5	45
y_10_	910.5069	910.5105	4	910.4741	36	40
y_11_	981.5446	981.5476	3.1	981.5112	34	37
y_12_	1038.5646	1038.5691	4.3	1038.5327	30.7	35
y_13_	1125.5952	1125.6011	5.2	1125.5647	27.1	32
y_14_	1254.6443	1254.6437	0.5	1254.6073	29.5	29
y_15_	1311.6582	1311.6652	5.3	1311.6288	22.4	28
y_16_	1424.7063	1424.7128	4.6	1408.6815	—	—

## 4. Discussion

### (a) Similarity between ostrich and alligator with the proposed ‘unique’ dinosaur peptides

Clearly, it would not be possible to disprove the hypothesis that the collagen sequences produced are indeed of dinosaur origin without sequencing an intermediate species between the extinct species in question and all of the extant taxa that had been sampled in the laboratory. Unfortunately, because ostrich was used, the species most commonly agreed as being earliest diverging from the extant bird lineage [23], this became no longer possible. If the original analyses had been carried out by comparison to a neognath bird (i.e. chicken), or at least a comparative species that left more basal extant taxa for others to sequence for phylogenetic support, this would have proven a testable hypothesis.

In our previous statement of concern [6] on the original reports [1,5], we pointed out that collagen is an ideal molecular target for assessing the risk of contamination. Despite its highly characteristic sequence motif, collagen is sufficiently variable for comparison between distinct taxa, if enough sequence is obtained. The results presented here show a complete match to all previously published peptides from both dinosaur specimens, indicating that this condition has not been met in either case. It may be that the proposed *Brachylophosaurus* sequence does differ from our ostrich sequence rather than a matching error. However, this would place *Brachylophosaurus* phylogenetically closer to chicken than ostrich [22], an unlikely scenario albeit with too few sequence changes to be of much value.

Cleland *et al.* [19] take this one step further and describe the matching of peptides from soft-tissues supposedly preserved in their *Brachylophosaurus* specimen. They do point out the observations of deamidation, although these will occur at high levels depending on extraction methods, likely more so in soft tissues than bone. In order to rule out cross-sample contamination, they present one peptide for ‘ostrich tubulin’ (AILVDLEPGTMDSVR) as being different from the *B. canadensis* (AVLVDLEPGTMDSVR), therefore reportedly attesting to the lack of contamination by ostrich and chicken proteins in the *B. canadensis* extractions (see Cleland *et al.* [19] and electronic supplementary material therein). However, what appears to have potentially been overlooked is that AVLVDLEPGTMDSVR (the reported ‘*B. canadensis* tubulin peptide’) is present in tubulin β-3 (NP_001074329.2) and β-5 (NP_001026183.1) chains in *Gallus*, tubulin β-5 (KFV86939.1) chain in *Struthio* (tubulin β-2 is the protein from which the above peptide they referred to originates), tubulin β-6 (XP_006269797.1) and β-4a (XP_006023414.1) chains in *Alligator* and several other tubulin chains among many other archosaur taxa. Likewise, AILVDLEPGTMDSVR (their ‘ostrich tubulin peptide’) is a tubulin β-2 sequence found not only in *Struthio* (KFV82917.1), but also in β-2 (NP_001004400.1), β-4 (NP_001026769.1) and β-7 (NP_990646.1) chains in *Gallus*, in β-2 (XP_006263527.3) and β-4b (XP_014462111.1) chains in *Alligator* and several other tubulin chains among many other archosaurs. All of the above matches are to the complete sequences, with 100% identity found from default protein BLAST searches.

### (b) Potential sources of contamination

There are three general sources of sample contamination, either (i) in the field, whether during the recovery or beforehand, (ii) during laboratory analyses or (iii) when curated and handled in museum/research collections. In consideration of the former [1]: the pes elements, tibia and fibula of *B. canadensis* were collected in 2006, whereas the femur was reported as being ‘protected under approximately 7 m of Judith River Formation sandstones' until 2007. It is unlikely that environmental contamination did arise at this point, but it cannot be ruled out. What is far more likely, given what we have observed with our own analyses, is laboratory cross-contamination of samples, coupled with long-term handling in collection environments (museum and research). It is not appropriate to list all specimens analysed within a particular laboratory or museum environment over a set time period (although this level of recording may indeed be necessary for ‘palaeoproteomics’ laboratories in the future). If the same laboratory has produced several publications relating to modern ostrich bone analysis, due diligence should note this as part of the samples' history. For example, Asara *et al.* [5] used modern ostrich bone, Schweitzer *et al.* [24] used modern emu bone, Schweitzer *et al.* [14] used modern ostrich and alligator, and modern ostrich blood vessels were used in the 2013 study [24].

The possibility of cross-contamination is typically dismissed on the grounds of sequence differences, and absence in sediment and analytical blanks. However, the latter alone cannot be considered appropriate grounds for such dismissal if the fossils are contaminated at a particular stage (e.g. handling, or sampling, for which both could differ between fossils and their sediment blanks), whereas the sequence differences are the primary focus of our study. In this regard, the authors in their original publication [1] only appeared to observe 30% sequence coverage of their ostrich bone collagen, despite typical values of greater than 60% (see electronic supplementary material, S1–S6). Given that we were able to observe high (76–80%) sequence coverages against even the chicken (I) collagen sequences, the lack of using closely related species in the searched database should not cause such a low coverage; even when a peptide ion score cut-off at the level suggested by Mascot for homology is used, sequence coverages remained more than twice that observed by Asara *et al*. [5].

In addition to this, at least a further 10 matches can be found in all three biological replicates with simple error tolerant searches (electronic supplementary material, S4–S6), along with a range of post-translational modifications expected with bone collagen, such as oxidations (M, P and K), deamidations (N and Q) and even those less commonly observed, such as glucosylgalactosyl modifications (K). Despite the overall poor sequence coverage for their modern ostrich collagen digest, one of the only two α 2(I) peptides that they did report (GLPGESGAVGPAGPIGSR) was homologous to the reported *T. rex* unique peptide (GLPGESGAVGPAGPPGSR). Nonetheless, our analyses (electronic supplementary material, S1–S6) demonstrate that their interpretation of the ostrich peptide sequence was likely incorrect, and that highly scoring peptide matches to the *T. rex* sequence are also observed from modern ostrich bone analysis. Phylogenetic analyses of such data (e.g. [4]) are entirely redundant, given that there are no confident differences in the amino acid sequences between the dinosaurs and the ostrich studied within the same laboratory. Separately, the same is true for the second set of peptides added for *B. canadensis* [22], which could independently derive solely from modern alligator. As such, the dangers of combining proteomics-derived datasets together from fossils should also be taken into account, particularly with phylogenetic reconstruction.

In 2011, San Antonio *et al*. [21] attempted to propose a preservation mechanism reported for the observed peptides that could potentially support the longevity of particular peptides. However, these could arguably be equally appropriate for sample cross-contamination and the peptides that survive within the laboratory environment. There are matches to other proteins, where Bern *et al.* [9] note that *Arachis hypogaea* (peanut) allergen appears out of place. However, in core facilities such as these, there is typically less control over previous runs without increasing costs. This is significant as we have also noticed carry-over from other samples submitted to our own core facility that are difficult to remove from LC–MS/MS of ever-increasing sensitivity. Bern *et al.* [9] pointed out that complete sequencing of ostrich collagen would help dispel one contamination scenario. Here, we have shown that even partial sequencing of ostrich collagen is enough to bring the findings for both dinosaur sequences into serious doubt. Given that modern ostrich (as well as alligator [14]) continued to be used as reference material ([5,19]), this is not an unrealistic speculation, but only the records of the laboratory itself could confirm the movement of the modern material throughout the various laboratories.

## 5. Conclusion

This report makes no attempt to address what the structures proposed by Schweitzer *et al.* [1] derive from, but emphasizes that the proteomic results may still be found to derive from laboratory contamination. With direct sequencing of biomolecules (DNA or protein), determination of whether sequences differ from those of all extant taxa taken into any of the laboratory environments should be a necessity with specimens of such antiquity. Although future analyses may reveal the survival of biomolecules of such antiquity, the fact that no other research groups have done so in the past decade since the 2007 study is itself informative. Our results suggest that cross-contamination should not be so readily dismissed as the likely source of collagen matched in earlier studies [5,14], thereby yielding the false-positive results for supposed dinosaur-derived collagen. The most recent 2017 study [22] does not find their unique dinosaur peptide (which we show as producing a good match for the homologous ostrich peptide) and entirely matches alligator. Yet in both studies, the levels of deamidation were suspiciously low, which can be used to detect recent contamination [25]. Hence, we urge that appropriate measures to test for endogeneity should remain an important part of the scientific process. The axiom that extraordinary claims require extraordinary evidence still stands and the case for dinosaur proteins is clearly no exception.

## Supplementary Material

Table S1

## Supplementary Material

Table S2

## Supplementary Material

Table S3

## Supplementary Material

Table S4

## Supplementary Material

Table S5

## Supplementary Material

Table S6

## Supplementary Material

Supplementary Material_PROCB2

## Data accessibility

The raw data are available on the PRIDE proteomics data repository as ‘Ostrich bone proteome’ (accession PXD006360; doi:10.6019/PXD006360) [26].
