# Supplementary material for: A fossil protein chimera; difficulties in discriminating dinosaur peptide sequences from modern cross-contamination
Source: Proc Biol Sci. 2017 May 31;284(1855):20170544. doi: 10.1098/rspb.2017.0544 (PMC5454271; doi:10.1098/rspb.2017.0544)
Supplement: Supplementary Material_PROCB2 [file rspb20170544supp7.docx]

**Supplementary Material**

**ESM file 1 - Table S1 – Standard LC-MS/MS search of ostrich CC1254 against SwissProt filtered on chicken (*Gallus gallus*), *Tyrannosaurus* and *Brachylophosaurus*.**

**ESM file 2 - Table S2 – Standard LC-MS/MS search of ostrich CC507 against SwissProt filtered on chicken (*Gallus gallus*), *Tyrannosaurus* and *Brachylophosaurus*.**

**ESM file 3 - Table S3 – Standard LC-MS/MS search of ostrich UM902 against SwissProt filtered on chicken (*Gallus gallus*), *Tyrannosaurus* and *Brachylophosaurus*.**

**ESM file 4 - Table S4 – Error tolerant LC-MS/MS search of ostrich CC1254 against SwissProt filtered on chicken (*Gallus gallus*), *Tyrannosaurus* and *Brachylophosaurus*.**

**ESM file 5 - Table S5 – Error tolerant LC-MS/MS search of ostrich CC507 against SwissProt filtered on chicken (*Gallus gallus*), *Tyrannosaurus* and *Brachylophosaurus*.**

**ESM file 6 - Table S6 – Error tolerant LC-MS/MS search of ostrich UM902 against SwissProt filtered on chicken (*Gallus gallus*), *Tyrannosaurus* and *Brachylophosaurus*.**

**The raw data is available on the PRIDE proteomics data repository as ‘Ostrich bone proteome’ (accession PXD006360; DOI: 10.6019/PXD006360).**
